# Identifying pregnancies in routinely collected health data: a scoping review of methods

**DOI:** 10.1186/s12911-026-03423-2

**Published:** 2026-04-18

**Authors:** Paolo Mazzone, Rose Higgins, Siobhan O’Connor, Jenny Myers, Tjeerd van Staa, Victoria Palin

**Affiliations:** 1 Maternal and Fetal Health Research Centre, The University of Manchester, Manchester, UK; 2 Bennet Institute for Applied Data Science, University of Oxford, Oxford, UK; 3 Florence Nightingale Faculty of Nursing, King’s College London, London, UK; 4 Centre for Health Informatics, The University of Manchester, Manchester, UK

**Keywords:** Pregnancy, Medical record linkage, Medical records systems, Algorithm, Electronic health records, Routinely collected health data

## Abstract

**Background:**

To map and describe the methods used to identify pregnancy episodes in routinely collected health data, to report validation practices and assess the transparency and reusability of methods.

**Methods:**

This study followed the Preferred Reporting Items for Systematic Reviews and Meta-Analyses for Scoping Reviews (PRISMA-ScR) guidelines. MEDLINE (Ovid), EMBASE (Ovid), OpenGrey, and Google Scholar were searched without time restrictions. Reference lists of relevant studies and reviews were screened for additional citations. All studies that utilised routinely collected health data to identify pregnancy episodes were included. Search results were imported into a reference management tool with duplicates removed. Title screening was conducted by one author. Two authors reviewed a subset (10%) of abstracts, with inter-rater agreement above 90% justifying the remainder of abstract review to be conducted by one author. This process was repeated at full-text review and during data extraction using a pre-piloted form.

**Results:**

From 5,859 records screened, 31 studies were included. 29 used rule-based backward-looking algorithms anchored to outcome codes and 2 used forward looking logic from early pregnancy makers. Nine studies incorporated hierarchical logic to estimate pregnancy start date and 19 introduced biologically plausible gaps between outcomes to mitigate misclassification. 15 studies conducted direct validation using chart review with inconsistent reporting of algorithm sensitivity, specificity, and PPV. While 22 studies shared code lists, only three provided reusable code.

**Conclusions:**

Future efforts should prioritise open-source algorithms, standardised validation protocols, and collaboration with clinical experts to ensure generalisability, reproducibility, and clinical relevance.

**Supplementary Information:**

The online version contains supplementary material available at 10.1186/s12911-026-03423-2.

## Background

Adverse maternal outcomes such as hypertensive disorders, preterm birth, stillbirth, severe maternal morbidity and maternal mortality remain major public health concerns across high, middle and low-income settings [1–3]. Pregnancy-related research relies on routinely collected health data as pregnant people are commonly excluded from clinical trials due to ethical and safety concerns [4]. Robust, population-level data are therefore required to provide evidence on maternal care pathways and risk of adverse maternal, fetal and offspring outcomes across diverse populations. This is particularly important considering that the risk of adverse outcomes is associated with pre-existing conditions such as psychiatric disorders [5], epilepsy [6] and diabetes [7], and that these risks are further compounded by additional demographic factors such as age [8, 9], ethnicity [10], and socioeconomic deprivation [11].

However, developing robust, population-level data is challenging. Variations in healthcare delivery and diagnostic coding practices, both nationally and internationally [12], mean that researchers have employed different methodologies and approaches to identify pregnancies in routinely collected health data [13]. These approaches vary in complexity, comprehensiveness, and validity [14]. Some studies identify a pregnancy based on maternity appointments and pregnancy outcomes [15, 16] yet they might unintentionally exclude pregnancies with no recorded outcome such as early loss [15] or restrict to live births [16]. Other studies have used gestational age at birth or last menstrual period to estimate the start of pregnancy [17], yet they have excluded pregnancies with less than two pregnancy markers to infer pregnancy start and end, such as antenatal visit and livebirth [17]. However, none of the studies previously mentioned assessed the validity of their approach.

Conversely, some researchers have clearly documented and validated their approach to identify pregnancy research using routinely collected health data around the world, across diverse coding systems including Read, Systematised Nomenclature of Medicine - Clinical Terms (SNOMED CT), and International Classification of Disease (ICD)-9/10 [18–26]. These demonstrate the feasibility of accurate pregnancy detection using rule-based algorithms that incorporate outcome dates, gestational age, and care markers. For instance, the Clinical Practice Research Datalink (CPRD) Pregnancy Register in the United Kingdom (UK) offers a curated source of pregnancy episodes based on clinically coded events and outcomes, and temporal logic [20, 25].

Despite the growing body of work in pregnancy identification, no comprehensive review has explored the breadth and depth of existing methodological approaches. This scoping review aimed to capture the diversity of approaches and synthesise them along with validation methods to inform future research using routinely collected health data in maternal and perinatal health.

### Primary objectives

1. To map existing methods used to identify pregnancy episodes using routinely collected health data in primary and secondary care.
2. To identify any reported performance of validated methods used for identifying pregnancies in routinely collected health data.

## Methods

This scoping review followed the Preferred Reporting Items for Systematic reviews and Meta-Analyses - Scoping Reviews (PRISMA-ScR) guidelines [27], and the protocol was pre-registered on the Open Science Framework (OSF) [https://osf.io/2ydqg].

### Search strategy

The search strategy was developed in accordance with the (Peer Review of Electronic Search Strategies) (PRESS) checklist [28]. All members of the research team contributed, with methodological input provided by an Information Specialist at the University of Manchester (Supplemental Material). Searches (Database inception-10/01/2026) were conducted in the MEDLINE (Ovid) and EMBASE (Ovid) databases, and grey literature was explored through the first 100 entries on both OpenGrey and Google Scholar. Reference lists of included studies and relevant systematic reviews were screened to identify additional eligible studies. No time restrictions were applied, but the review was limited to studies published in English. Conference abstracts were excluded.

### Study screening and selection

Eligible studies included those that used routinely collected health data to identify pregnancies. Only observational studies were included, encompassing data from community, primary, secondary, and tertiary care settings. Clinical trials were excluded unless they incorporated and validated methods of pregnancy identification using routine data. To qualify, studies were required to document their method of pregnancy identification, including the estimation of pregnancy start and end dates and/or the use of coded diagnostic indicators for pregnancy-related events or outcomes. Studies were excluded if their primary aim was not to develop a method for pregnancy identification using routinely collected health data, or if they relied on machine learning techniques which limit transferability due to ethical and disclosure concerns when using routinely collected health data. Search results were imported into the Rayyan [29], and duplicates removed. Title screening was conducted independently by one author (PM), with a liberal inclusion approach to minimise early-stage exclusion bias. A 10% subset of abstracts was independently reviewed by two authors (PM, SO); an inter-rater agreement exceeding 90% allowed the remainder of the abstracts to be screened by one reviewer. This process was repeated at the full-text screening stage, with disagreements resolved through discussion or, where necessary, arbitration by a third author (VP).

### Data extraction

Data extraction was undertaken using a form developed and piloted by authors (PM, RH, VP). Two authors (PM, RH) independently extracted data from a subset of included studies, with disagreements resolved through discussion or arbitration by a third author (VP). An inter-rater reliability above 90% allowed for the remaining data to be extracted by one author (PM). We extracted information from each study according to four pre-specified themes: (1) gestational age (GA) estimation methods, (2) algorithms used to determine pregnancy start and end dates, (3) validation approaches for the algorithms, and (4) transparency and reusability of the methods. Figures and tables were designed to provide an overview of key findings: a PRISMA flowchart outlines study selection, while a baseline characteristics table summarises study design, data source, geographic location, study period, and number of pregnancies. Detailed raw data tables are provided in the Supplementary Material.

### Data presentation and synthesis

A PRISMA flow diagram was used to present the study selection process. Data on pregnancy identification algorithms were presented in structured tables and descriptive summaries. The first objective was to map methods used to identify pregnancy episodes in routinely collected health data, including coding systems, and how gestational age, pregnancy start and end dates were estimated. The second objective summarised the accuracy of the identified algorithms, including performance metrics such as sensitivity, specificity, PPV, NPV, AUC, or concordance with reference standards where available. This review will also assess the transparency and reusability of methods used to identify pregnancies using routinely collected health data, such as publicly available code lists and variables used, algorithm logic and open-source coding.

## Results

**Fig. 1:**
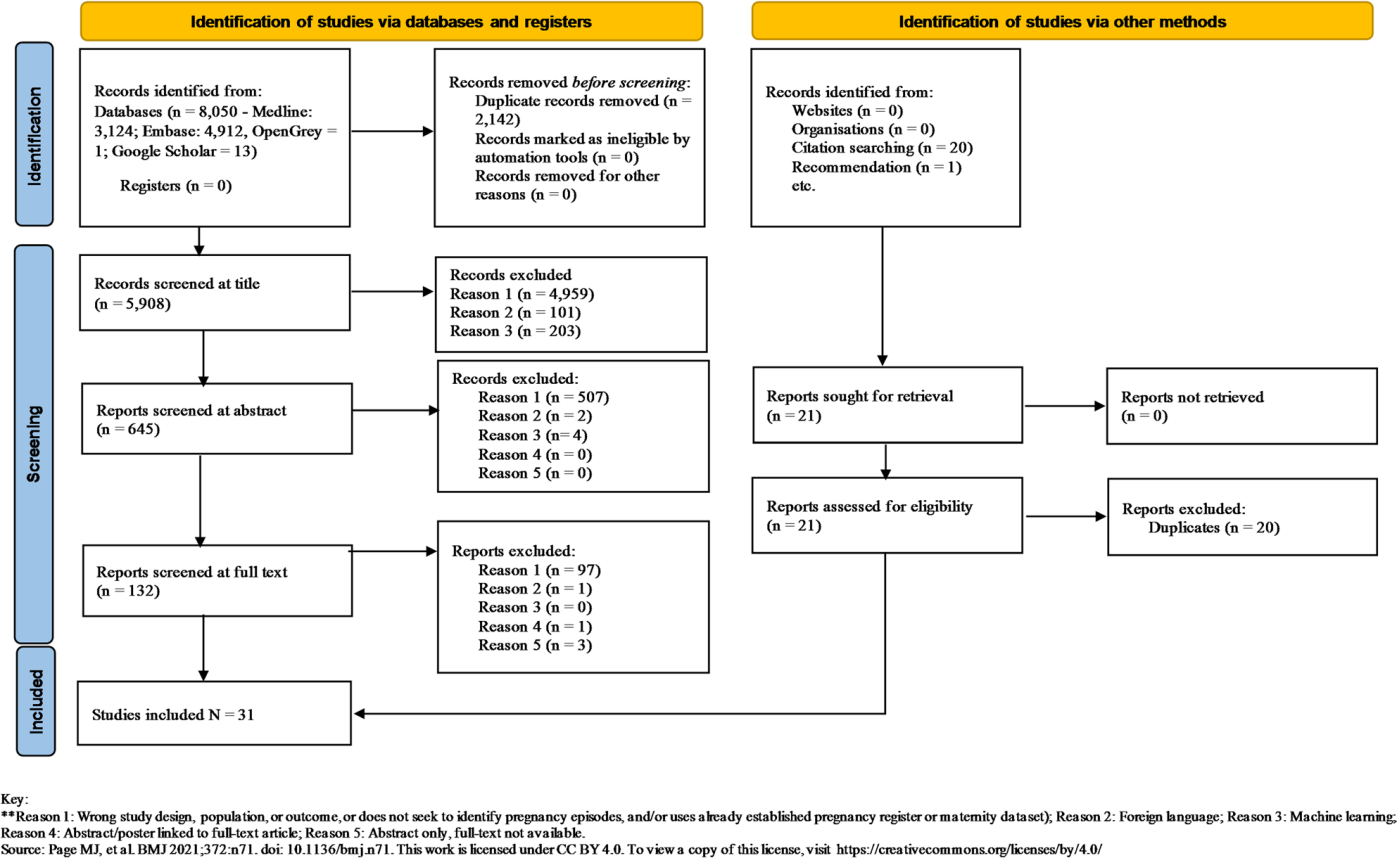
PRISMA 2020 flow diagram

This review identified a total of 5,859 papers for title, 646 for abstract, and 133 for full-text screening (Fig. 1). A total of 31 studies published between 2001 and 2025 were included in this review. Key findings are summarised in Tables 1, 2 and 3. Table 1 provides study characteristics and data sources, Table 2 summarises GA and pregnancy start and end-date estimation methods, and Table 3 reports validation approaches and reported metrics. Studies were conducted across the United States of America, Europe, and New Zealand, and utilised a range of data sources, including administrative/claims, electronic health records (EHRs), registries, and combinations thereof (Table 1). Information on baseline characteristics of pregnancies were reported inconsistently, with 14 studies providing maternal age [18, 20, 21, 23, 26, 30–38], two providing deprivation [33, 38], two providing pre-existing conditions [34, 36], and six providing ethnicity [21, 23, 26, 33, 34, 38]. Detailed algorithm specifications and individual study results are included in Supplementary Material (Tables 2, 3 and 4).

### Pregnancy identification methods

#### Data sources and code lists

Each specific data source used one or more clinical coding systems, such as ICD, Read, SNOMED-CT, Diagnosis Related Group (DRG), Healthcare Common Procedure Coding System (HCPCS), Logical Observation Identifiers Names and Codes (LOINC), Current Procedural Terminology (CPT), OPCS, or other country or database specific coding systems. These coding systems use a uniform language to systematically identify specific clinical activities, events or outcomes which can then be used to identify a range of pregnancy-related events and outcomes such as antenatal visits, pregnancy losses, or deliveries. Table 1 provides counts of studies for each country and for each data source.

**Table 1:** Baseline Study Characteristics^1^

Characteristic	Number of Studies (%)	References
Total Studies	31	
Geographic Distribution		
*United States of America*	20 (64.5)	[17, 18, 21–23, 26, 30, 32, 34, 36–46]
*United Kingdom*	7 (22.6)	[15–17, 20, 25, 35, 47]
*France*	1 (3.2)	[31]
*Germany*	1 (3.2)	[48]
*Norway*	1 (3.2)	[49]
*New Zealand*	1 (3.2)	[33]
*Korea*	1 (3.2)	[50]
Data Sources		
*Administrative or claims data*	14 (45.2)	[18, 26, 30–32, 34, 36, 39, 40, 43, 45, 46, 48, 50]
*EHR data*	12 (38.7)	[15, 16, 20– 23, 25, 35, 38, 42, 44, 47]
*Registry data*	1 (3.2)	[49]
*EHR + claims data*	2 (6.4)	[17, 33]
*EHR + administrative data*	2 (6.4)	[37, 41]

Notes: Percentages calculated from the 29 included studies; yet some studies employed multiple methods, hence percentages may exceed 100% in relevant categories

^1^Individual study characteristics and methods are provided in the Supplemental Material, Tables 2, 3 and 4

#### Overview of pregnancy identification algorithm logic

All studies used rule-based algorithms to identify pregnancy episodes. However, studies varied in their approaches to the identification of pregnancy start and end dates, gestational age (GA) estimation, and handling of overlapping or conflicting pregnancy outcome codes. Overall, methods comprised one or all of the following strategies but with varying complexity: (1) identification of pregnancy or outcome events to estimate: (a) pregnancy-start date, (b) pregnancy-end date, (c) GA; (2) application of temporal and hierarchical logic throughout the pipeline to define pregnancy episodes; (3) handling of overlapping episodes and conflict resolution; (4) validation of methods. Table 2 provides a summary of study approaches with specific examples provided in the subsequent sections of the results.

#### Gestational age estimation

Gestational age (GA) was obtained most commonly from specific GA codes (such as those indicating number of weeks gestation) or discharge/birth records (Table 2). When such data were missing, outcome‑specific defaults were used or GA was approximated from the delivery date alone. Hierarchical estimation integrated multiple sources in a defined order; one implementation prioritised linked birth records (live births) using the following hierarchy: (a) linked birth records; (b) estimated date of delivery (EDD); (c) ICD-10 GA codes; (d) Last menstrual period (LMP), and (e) imputed GA based on outcome type [17]. In data‑limited settings, GA estimation was not performed [40, 48].

#### Pregnancy start date estimation

Start dates were established using multiple methods (Table 2). Early-marker-based approaches defined start dates using the earliest or first pregnancy-related event within defined windows prior to the outcome date, such as a positive human chorionic gonadotropin (hCG), prenatal vitamin and ultrasound orders [44], or positive hCG and prenatal visits [40]. Another approach involved looking forward from early pregnancy markers for up to nine months to identify full-term to post-term deliveries [40].

However, the most common method involved back-calculation, subtracting GA data (obtained from clinical coding terminology such as ICD-10-CM Z3A codes - which are used in the United States of America to represent the weeks of gestation of a pregnancy) from a known pregnancy outcome date. Another approach involved imputing biologically plausible gestational start dates when GA codes and early pregnancy-marker data were unavailable, such as 273 days for term deliveries [34] or 70 days for termination of pregnancy [30], and where laboratory and testing data were available, start dates were inferred by estimating the last menstrual period (LMP) from the observed timing of the first and second antenatal testing [33]. Hierarchical assignment combined multiple sources in descending priority, for example, one study hierarchically assigned pregnancy start dates based on the following hierarchy: estimated date of conception (EDC), LMP, antenatal GA, delivery GA, or biologically plausible imputed values when all other data were missing [25].

#### Pregnancy end date estimation

Outcome codes defined the pregnancy end date across all settings (Table 2). Temporal plausibility was enforced via minimum inter‑pregnancy gaps tailored to outcome types, such as a window of ≥ 90 days [45], ≥ 28 weeks [31] or ≥ 120 days for live births and ≥ 42 days for other outcomes such as miscarriage [18]. To prevent over‑counting, code‑cluster logic collapsed multiple outcome codes occurring in close succession into a single episode [15, 33] and implausible duplicates were excluded in favour of the most likely valid episode [17, 23, 49]. Detailed conflict‑resolution rules prioritised the earliest outcome within category‑specific windows, for example, 60 days for terminations/miscarriages and 210 days for live or stillbirths and then assigning any subsequent outcome codes after those windows to new pregnancies [15]. Another study utilised hierarchical logic for early pregnancy loss codes, with the type of loss determined in order of priority: ectopic, termination, miscarriage, probable termination, molar, unspecified loss, and blighted ovum [25].

#### Handling of overlapping or conflicting episodes

Overlaps and conflicts were handled using biological minimum gaps, code‑clustering and exclusion or review pathways (Table 2). Admissions with outcome codes occurring within six weeks were treated as a single pregnancy, using the earliest admission in the cluster to derive start and end dates [33]. Two studies flagged overlapping outcome codes for further inspection rather than excluding them [20, 25], another approach was to exclude episodes with conflicting information outright [32]. Approaches to conflict handling were not specified in some cases [37, 41, 42, 44] and one restricted to the first pregnancy for each person [40].

**Table 2:** Summary Results by Pregnancy Identification Methods^2^

Methods	Number of Studies (%)	References
Overarching algorithm logic		
*Backward-looking*	29 (93.6)	[15–18, 20–23, 25, 26, 30–39, 41–43, 45–50]
*Forward-looking*	2 (6.4)	[40, 44]
Gestational age estimation		
*GA codes*	18 (58.1)	[17, 18, 20, 22, 23, 25, 26, 30–32, 34, 36, 38, 41, 43, 45, 49, 50]
*Imputed outcome-specific defaults when GA data were missing*	10 (32.3)	[20, 25, 31, 32, 34, 37, 39, 41, 43, 49]
*Approximated based on delivery date alone*	8 (25.8)	[15, 16, 21, 33, 35, 42, 44, 46]
*Hierarchical estimation*	3 (9.7)	[17, 41, 47]
*Not estimated*	2 (6.4)	[40, 48]
Start date estimation methods		
*Early-marker based*	5 (16.2)	[15, 16, 35, 40, 44]
*Back-calculation by subtracting GA or imputing biologically plausible start date from outcome date*	26 (83.9)	[15–18, 20–23, 25, 26, 30–39, 41, 43, 45, 46, 48, 49]
*Hierarchical*	11 (35.5)	[17, 20, 22, 25, 26, 30, 35, 41, 47, 49, 50]
End date definition methods		
*Outcome anchored*	31 (100.0)	[15–18, 20–23, 25, 26, 30–50]
Conflict and overlap handling		
*Minimum gap requirements*	19 (61.3)	[15–18, 20, 22, 23, 25, 26, 30, 31, 34, 36, 38, 39, 43, 45, 47–50]
*Overlapping pregnancies flagged*	2 (6.4)	[15, 33]
*Excluded duplicates*	1 (3.2)	[32]
*Code clusters and hierarchical logic*	6 (19.4)	[15, 20, 25, 33, 35, 46, 47, 50]
*Restricted to first pregnancy only*	1 (3.2)	[40]
*Not defined*	5 (16.2)	[21, 37, 41, 42, 44]

Notes: Percentages calculated from the 29 included studies; yet some studies employed multiple methods, hence percentages may exceed 100% in relevant categories

^2^Individual study characteristics and methods are provided in the Supplemental Material, Tables 2, 3 and 4

### Validation approaches

Validation strategies included indirect benchmarking (comparing outcome distributions with national statistics, registries, surveys and prior estimates) and direct validation via clinical review of chart abstraction or inspection. The reported performance of pregnancy algorithms ability to identify outcomes were measured using positive predictive value, negative predictive value, sensitivity, specificity and agreement‑based metrics such as percentage agreement and Cohen’s Kappa. Table 3 provides an outline of these findings, with study specific metrics presented in the Supplemental Material (Tables 2, 3 and 4).

#### Transparency and reusability

Twenty studies published code lists [17, 18, 20, 22, 23, 25, 26, 30–34, 36–39, 42, 45, 46, 49]; however, cross‑mapping to alternative coding systems and documentation of clinical input were inconsistent. Algorithm reproducibility was generally limited to text and illustrative descriptions of logic and filtering rules, with only three studies providing reusable code: one provided a full code outline [18], one provided a partial extract of pseudo-code for episode start and outcome identification [17], and one provided code relating to gestational week estimation [23].

**Table 3:** Algorithm Performance Summary^3^

	Number of Studies (%)	References
**Validation approaches**		
*Direct validation*	15 (48.4)	[17, 21–23, 26, 32, 35, 37, 40–42, 44, 46, 48, 49].
*Indirect validation*	12 (38.7)	[15, 16, 18, 20, 25, 31, 33, 36, 38, 39, 45, 47]
*No validation reported*	4 (12.9)	[30, 34, 43, 50]
Performance metrics reported		
*Positive Predictive Value (PPV)*	8 (25.8)	[21, 25, 32, 38, 42, 46, 47, 49]
*Sensitivity*	4 (12.9)	[21, 25, 38, 42]
*Specificity*	2 (6.4)	[38, 44]
*Negative Predictive Value (NPV)*	2 (6.4)	[38, 49]
*Cohen’s Kappa*	2 (6.4)	[15, 49]
*Percentage agreement*	9 (29.1)	[17, 22, 23, 26, 31, 37, 40, 41, 48]
*No performance metrics*	12 (38.7)	[16, 18, 20, 30, 33–36, 39, 43, 45, 50]

Notes: Percentages calculated from the 29 included studies; yet some studies employed multiple methods, hence percentages may exceed 100% in relevant categories

^3^Individual study characteristics and methods are provided in the Supplemental Material, Tables 2, 3 and 4

## Discussion

Despite broad reliance on rule-based algorithms across the 29 included studies, differences in algorithmic logic, data source characteristics, and validation practices highlight the challenges in developing robust methods for pregnancy identification. These findings have important implications for downstream maternal and fetal health research, including the need for transparent reporting to ensure interoperable algorithm development, rigor and reproducibility.

### Data source and coding system heterogeneity

One source of methodological variation is a result of data source diversity and underlying clinical coding systems. Studies using EHRs [15, 16, 20–23, 25, 35, 38, 42, 44, 47] can leverage granular clinical detail such as gestational age, and pregnancy complications and outcomes that can contribute to more precise dating and classification. Claims/administrative data [18, 26, 30–32, 34, 36, 39, 40, 43, 45, 46, 48, 50] optimise coverage but may miss pregnancy events that do not generate billable activity, including early losses or complications without intervention.

Availability of specific GA codes such as ICD‑10‑CM Z3A, facilitates back‑calculation of pregnancy start [17, 18, 20, 23, 25, 26, 30–32, 34, 36, 38, 41, 43, 45], whereas settings without these data require imputation or delivery‑date approximation [15, 16, 21, 33, 35, 42, 44, 46]. Moreover, although the majority of studies provided code lists [17, 18, 20, 22, 23, 25, 26, 30–34, 36–39, 42, 45–47, 49, 50], few mapped these across clinical coding systems or detailed clinician involvement, constraining generalisability and replicability. This is particularly important for pregnancy research where international comparisons are increasingly important for understanding global maternal health outcomes.

Multi-source linkage, maternity apps, patient portals, and consented registries can improve comprehensiveness of data capture. However, these sources require robust consent, governance, linkage, and equity safeguards to avoid over-representing digitally connected or those from less deprived groups.

### Algorithmic logic

The dominance of backward-looking algorithms [18, 23, 30–39, 43, 45–50] reflects both the availability and perceived reliability of outcome codes. While this approach simplifies logic and mitigates some uncertainties associated with incomplete antenatal data, it introduces survivorship bias. Survivorship bias occurs when algorithms only capture pregnancies that “survive” to produce a recorded outcome, systematically excluding early pregnancy losses, miscarriages, and terminations that may not generate complete outcome codes. This limitation can impact research validity and prevents comprehensive research into pre- and inter-conceptual health, particularly for women experiencing recurrent pregnancy losses, and limits studies of risk factors for pregnancy loss, early pregnancy complications, and effectiveness of interventions for high-risk pregnancies [51]. For questions concerning early‑pregnancy safety or recurrent loss, algorithms should therefore incorporate early‑marker capture where feasible, or at minimum prespecify sensitivity analyses that vary look‑back and look‑forward windows and imputation schemes to characterise the direction and magnitude of potential survivorship bias.

### Start‑date estimation, end‑date definition and gestational age: an end‑to‑end pipeline

The variation in algorithm logic, such as how algorithms handled start date and GA estimation reflects fundamental challenges in pregnancy identification research. Algorithm design choices about how to estimate GA, and pregnancy start, and end dates are not interchangeable and have direct effects on eligibility, exposure timing and risk attribution. These decisions have significant implications for study validity and cross-study comparability.

Early‑marker strategies are clinically intuitive for early‑pregnancy safety questions but depend on timely recording; delays or sparse capture can misdate conception and widen uncertainty around exposure windows consistent with early‑marker implementations [15, 16, 40, 44] and inference of last menstrual period from the timing of first and second antenatal testing [33]. Biologically plausible defaults allow algorithms implemented in data with sparse event coding to apply a start-date without early marker data. However, this does not account for variations in gestational length, for example, 273 days for term or 70 days for termination [30, 34]. Back‑calculation from coded GA is operationally simple and can be precise when coding is complete [18, 23, 30–34, 36–39, 43, 45, 46, 48–50]; however, it may silently misclassify timing when GA is missing or miscoded, and it inherits any systematic coding biases. Hierarchical approaches (for example, estimated date of conception, last menstrual period, antenatal gestational age, delivery gestational age, then imputed values) mitigate single‑source weaknesses only if source order reflects clinical credibility and is justified a priori [17, 20, 22, 25, 26, 30, 35, 41, 47, 49]. Accordingly, source order, temporal windows and defaults should be explicit, clinically justified and stress‑tested; sensitivity analyses should vary look‑back/look‑forward windows and reorder sources to quantify robustness of inclusion and timing.

End‑date logic and conflict handling embed clinical and coding assumptions that directly alter pregnancy counts and inter‑pregnancy intervals. Minimum‑gap and category‑specific conflict windows reduce over‑counting (examples include ≥ 90 days, ≥ 28 weeks, and ≥ 120/42 days; 60‑ and 210‑day windows [15–18, 20–23, 25, 26, 30–50]. Shorter windows risk fragmenting a single biological episode. Longer windows risk collapsing adjacent pregnancies and depressing event rates. Both effects complicate cross‑study comparability where coding intensity differs. Code‑cluster collapsing and duplicate resolution improve parsimony but may obscure clinical complexity in high‑utilisation pathways [15, 17, 23, 33, 49]. Early pregnancy loss categorisation followed hierarchies (ectopic, termination, miscarriage, probable termination, molar, unspecified loss, blighted ovum) [25]. Operational strategies for overlaps included treating admissions with outcome codes within six weeks as a single episode anchored to the earliest admission [33], flagging overlaps for review rather than exclusion [20, 25], excluding episodes with conflicting information [32], and restricting analysis to the first pregnancy per person where approaches were otherwise unspecified [40]. To support transferability and interpretation, studies should report the clinical rationale for chosen thresholds, provide episode‑count sensitivity to window changes, and quantify the impact of merge/flag/exclude decisions on retained episodes, inter‑pregnancy interval distributions and outcome assignment.

GA estimation underpins exposure assignment, trimester‑specific outcomes and eligibility, yet code‑based GA is vulnerable to missingness and miscoding. Approximation of GA based on outcome type is widely available but can misclassify pregnancy start date when outcome specific defaults are used. This is particularly important for various contexts, such as identifying pregnancy start for medication exposure, or gestational age for stages of fetal development or to identify term or pre-term deliveries [15–18, 20–23, 25, 26, 30–32, 34, 36, 38, 41, 43, 45]. Hierarchies and fallback rules should therefore be prespecified, with cross‑source discrepancy audits where multiple GA sources co‑exist (for example, cross‑tabulating code‑based versus discharge‑based GA), and bounding analyses that vary defaults and repeat estimation using alternative GA sources to demonstrate stability of effect estimates and eligibility definitions.

### Handling of overlapping episodes: methodological challenges and implications

Overlapping episodes occur when the ordering or spacing of a series of pregnancy or delivery codes, or even episode counts, renders episode boundaries unclear. For example, antenatal codes shortly after a delivery code followed by additional delivery codes may reflect either two pregnancies or miscoded/implausible events coded post‑delivery. This represents a methodological design problem for pregnancy algorithms. Studies using ICD‑10‑CM with GA codes such as Z3A were more likely to implement hierarchical approaches and explicitly address overlaps, as precise GA information permits finer conflict resolution [18, 23, 30–36, 38, 39, 41, 43, 46, 48–50]. The choice of overlap handling strategy has significant implications for end-users of pregnancy research. Merging events within a short window reduces double‑counting but risks collapsing adjacent pregnancies. Exclusion yields clean cohorts but can remove genuine clinical sequences. Hierarchical resolution (prioritising clinically plausible outcome sequences) preserves episodes at the cost of model complexity. Flag‑for‑review can improve accuracy where feasible but may be impractical at scale. Restricting to the first pregnancy simplifies analysis but can bias estimates in high‑parity populations. Algorithms should: (i) publish decision trees for overlap resolution (including any early‑loss hierarchies); (ii) justify overlap and conflict windows clinically and stress‑test them according to alternative window strategies; and (iii) report standard diagnostics such as the proportion of episodes flagged, merged and excluded, and the resultant change in total episode counts, inter‑pregnancy intervals and outcome assignment.

### Validation practices: feasibility and methodological considerations

Validation practices in the included literature ranged from indirect benchmarking against external sources to direct chart or clinical inspection. Where access is limited to anonymised or aggregated data without clinical connectivity, traditional chart review may be impracticable; in such settings, indirect validation should be complemented by internal digital checks (for example, cross‑source consistency between primary and secondary care records, procedure–outcome verification, and temporal cross‑validation of related events), and residual uncertainty should be stated explicitly. Where clinical connectivity permits record‑level review, protocols should prespecify the sampling frame and reference standard, ensure reviewer blinding to algorithm outputs where feasible, and report appropriate agreement metrics (positive/negative predictive values, sensitivity, specificity, percentage agreement, Cohen’s Kappa).

### Transparency and reusability: supporting scientific understanding and improvement

While open science practices have improved, the reproducibility of pregnancy algorithms remains limited. Only three studies provided any form of reusable code [17, 18, 23], and two of these were partial or limited in scope [18, 23]. Although it is important to note that truly “ready-to-implement” analytic code is not likely to be possible in practice, as databases have different structures, field names, and data formats across healthcare systems, transparency in methods serves multiple purposes beyond simple reproducibility. Transparency and open science practices supports understanding of methodological approaches, enables accurate critique and validation of findings, and can lead to improvement in algorithmic approaches. The publication of code lists, methodological descriptions, and reusable elements such as analytical code and validation frameworks supports scientific reuse and rigor, even when full code implementation requires adaptation to local data structures.

### Strengths and limitations

This scoping review represents the first comprehensive review to explore the breadth and depth of existing methodological approaches and validation techniques in pregnancy identification algorithms. By synthesising 31 studies representing diverse healthcare systems, data sources, and methodological approaches, we have identified key patterns and methods in how pregnancy episodes are identified, validated, and handled across different clinical and research contexts. We have mapped and described methodological approaches, gaps and areas for improvement, whilst also considering the impact of transparency and reproducibility practices. However, conducting a scoping review does bring some inherent limitations such as a focus on breadth rather than depth of information, no formal comparison of methods, and no assessment of the methodological quality or risk of bias of the included studies. However, in-lieu of a formal quality assessment, we have described the potential risks introduced by the most common approaches currently used for identifying pregnancy episodes, including survivorship bias, under-reporting of early losses, and inconsistent handling of overlapping episodes. Another potential limitation is that one author (PM) screened at title stage; this could increase the chance of excluding relevant papers. However, it is generally considered acceptable that this initial screening is undertaken by one person [52].

### Future work

Collaboration with clinical experts, informaticians, and data custodians is important to ensure algorithms are methodologically robust and clinically relevant, particularly in addressing the challenges of overlapping episodes and incomplete pregnancy data. This would improve data source reliability and subsequent policy relevant research for improved clinical guidance and decision making. Furthermore, validation of methods should be standard practice, alongside open-source codelists, documentation, and algorithms that can be more easily adapted across different healthcare information systems and data structures to improve standards of eHealth; whilst recognising that true “plug-and-play” implementation is not feasible given database heterogeneity.

## Supplementary Information

Below is the link to the electronic supplementary material.

Supplementary Material 1

## Data Availability

No datasets were generated or analysed during the current study.

## Declarations

### Copyright

For the purpose of open access, the author has applied a CC-BY public copyright licence to any Author Accepted Manuscript version arising from this submission.
