# Supplementary material for: Identifying pregnancies in routinely collected health data: a scoping review of methods
Source: BMC Med Inform Decis Mak. 2026 Apr 18;26:196. doi: 10.1186/s12911-026-03423-2 (PMC13227625; doi:10.1186/s12911-026-03423-2)
Supplement: Supplementary file 1 — Supplementary Material 1 [file 12911_2026_3423_MOESM1_ESM.docx]

**Table of Contents**

[Table 1. Preferred Reporting Items for Systematic reviews and Meta-Analyses extension for Scoping Reviews (PRISMA-ScR) Checklist 2](#_Toc219285640)

[Search Terms and Strategy 4](#_Toc219285641)

[Table 2. Pregnancy Identification Algorithms - Electronic Health Records 6](#_Toc219285642)

[Table 3. Pregnancy Identification Algorithms - Claims Data 8](#_Toc219285643)

[Table 4. Pregnancy Identification Algorithms - Registry Data and Combination of Health Records 10](#_Toc219285644)

[References 12](#_Toc219285645)

# Table 1. Preferred Reporting Items for Systematic reviews and Meta-Analyses extension for Scoping Reviews (PRISMA-ScR) Checklist

| **SECTION** | **ITEM** | **PRISMA-ScR CHECKLIST ITEM** | **REPORTED ON PAGE #** |
| --- | --- | --- | --- |
| **TITLE** | | | |
| Title | 1 | Identify the report as a scoping review. | 1 |
| **ABSTRACT** | | | |
| Structured summary | 2 | Provide a structured summary that includes (as applicable): background, objectives, eligibility criteria, sources of evidence, charting methods, results, and conclusions that relate to the review questions and objectives. | 2 |
| **INTRODUCTION** | | | |
| Rationale | 3 | Describe the rationale for the review in the context of what is already known. Explain why the review questions/objectives lend themselves to a scoping review approach. | 3 |
| Objectives | 4 | Provide an explicit statement of the questions and objectives being addressed with reference to their key elements (e.g., population or participants, concepts, and context) or other relevant key elements used to conceptualize the review questions and/or objectives. | 4 |
| **METHODS** | | | |
| Protocol and registration | 5 | Indicate whether a review protocol exists; state if and where it can be accessed (e.g., a Web address); and if available, provide registration information, including the registration number. | 5 |
| Eligibility criteria | 6 | Specify characteristics of the sources of evidence used as eligibility criteria (e.g., years considered, language, and publication status), and provide a rationale. | 5 |
| Information sources* | 7 | Describe all information sources in the search (e.g., databases with dates of coverage and contact with authors to identify additional sources), as well as the date the most recent search was executed. | 5 |
| Search | 8 | Present the full electronic search strategy for at least 1 database, including any limits used, such that it could be repeated. | Supplemental Material Page 4-5 |
| Selection of sources of evidence† | 9 | State the process for selecting sources of evidence (i.e., screening and eligibility) included in the scoping review. | 5-6 |
| Data charting process‡ | 10 | Describe the methods of charting data from the included sources of evidence (e.g., calibrated forms or forms that have been tested by the team before their use, and whether data charting was done independently or in duplicate) and any processes for obtaining and confirming data from investigators. | 5-6 |
| Data items | 11 | List and define all variables for which data were sought and any assumptions and simplifications made. | Supplemental Material Page 6-10 |
| Critical appraisal of individual sources of evidence§ | 12 | If done, provide a rationale for conducting a critical appraisal of included sources of evidence; describe the methods used and how this information was used in any data synthesis (if appropriate). | 5-6 |
| Synthesis of results | 13 | Describe the methods of handling and summarizing the data that were charted. | 5-6 |
| **RESULTS** | | | |
| Selection of sources of evidence | 14 | Give numbers of sources of evidence screened, assessed for eligibility, and included in the review, with reasons for exclusions at each stage, ideally using a flow diagram. | 7-13 |
| Characteristics of sources of evidence | 15 | For each source of evidence, present characteristics for which data were charted and provide the citations. | 7-13 and Supplemental Material Page 6-10 |
| Critical appraisal within sources of evidence | 16 | If done, present data on critical appraisal of included sources of evidence (see item 12). | 7-13 |
| Results of individual sources of evidence | 17 | For each included source of evidence, present the relevant data that were charted that relate to the review questions and objectives. | 7-13 and Supplemental Material Page 6-10 |
| Synthesis of results | 18 | Summarize and/or present the charting results as they relate to the review questions and objectives. | 7-13 and Supplemental Material Page 6-10 |
| **DISCUSSION** | | | |
| Summary of evidence | 19 | Summarize the main results (including an overview of concepts, themes, and types of evidence available), link to the review questions and objectives, and consider the relevance to key groups. | 14-17 |
| Limitations | 20 | Discuss the limitations of the scoping review process. | 17-18 |
| Conclusions | 21 | Provide a general interpretation of the results with respect to the review questions and objectives, as well as potential implications and/or next steps. | 17-18 |
| **FUNDING** | | | |
| Funding | 22 | Describe sources of funding for the included sources of evidence, as well as sources of funding for the scoping review. Describe the role of the funders of the scoping review. | 19 |

JBI = Joanna Briggs Institute; PRISMA-ScR = Preferred Reporting Items for Systematic reviews and Meta-Analyses extension for Scoping Reviews.

* Where *sources of evidence* (see second footnote) are compiled from, such as bibliographic databases, social media platforms, and Web sites.

† A more inclusive/heterogeneous term used to account for the different types of evidence or data sources (e.g., quantitative and/or qualitative research, expert opinion, and policy documents) that may be eligible in a scoping review as opposed to only studies. This is not to be confused with *information sources* (see first footnote).

‡ The frameworks by Arksey and O’Malley (6) and Levac and colleagues (7) and the JBI guidance (4, 5) refer to the process of data extraction in a scoping review as data charting*.*

§ The process of systematically examining research evidence to assess its validity, results, and relevance before using it to inform a decision. This term is used for items 12 and 19 instead of "risk of bias" (which is more applicable to systematic reviews of interventions) to include and acknowledge the various sources of evidence that may be used in a scoping review (e.g., quantitative and/or qualitative research, expert opinion, and policy document).

*From:* Tricco AC, Lillie E, Zarin W, O'Brien KK, Colquhoun H, Levac D, et al. PRISMA Extension for Scoping Reviews (PRISMAScR): Checklist and Explanation. Ann Intern Med. 2018;169:467–473. doi: 10.7326/M18-0850.

# Search Terms and Strategy

**EMBASE & MEDLINE Search Terms**

**Population**

1. exp *Pregnant People/ or exp *Mothers/ or exp *Adolescent Mothers/ or (pregnant people or pregnancy or pregnant person$ or birthing people or birthing person$).mp.
2. exp *Pregnancy/
3. exp *Maternal Health Services/
4. (Maternity or Antenatal or maternal or perinatal or pregnancy or maternity care or antenatal care or perinatal care or pregnancy care or pregnant care).mp.
5. exp *Perinatal Care/

**Intervention/Exposure**

1. exp Medical Record Linkage/ or (medical records or medical record linkage).mp.
2. (health$ record$ or health$ data$ or medical record$ or medical data$ or routin$ health$ records or routin$ health$ data$ or routin$ collected health$ data$ or routin$ collected health$ records or routin$ collected administrative health$ data$ or routin$ collected administrative health$ records or administrative health$ data$ or administrative health$ records or administrative data$ or administrative records or electronic patient records or digital records or hospital episode statistic$ or hospital record$ or maternity episode or pregnancy episode or pregnancy-episode or national data$ or health data$ or linked administrative data$ or longitudinal data$ or electronic health records or electronic health$ data$ or electronic medical record$ or electronic patient record$).mp.
3. exp Medical Records Systems, Computerized/ or exp Electronic Health Records/ or exp Hospitalization sn/ [Statistics & Numerical Data]
4. exp *Registries/
5. exp *"Information Storage and Retrieval"/

**Outcome**

1. ((pregnan$ adj identif$) or (estimated gestation$ start or estimated gestation$ length or estimated conception or estimated gestation or pregnancy start or pregnancy end or gestation$ start or gestation$ end or gestation$ length or estimated pregnancy or estimated delivery or estimated birth or estimated delivery date or estimated birth date or EDD or gestation$ period or pregnancy period) or (pregnancy adj date) or (pregnancy adj1 date)).mp.
2. (linking or linkage or data-linking or data-linkage or data linking or data linkage).mp.
3. (mother-baby link or mother baby link or mother-baby linkage or mother baby linkage or mother baby linking or mother-baby linking).mp.
4. (mother baby pairs or mother-baby pairs or mother child pairs or mother-child pairs or mother baby dyads or mother-baby dyads or mother child dyads or mother-child dyads or mother baby cohort or mother-baby cohort or mother child cohort or mother-child cohort).mp.
5. (deterministic algorithm or probabilistic algorithm or deterministic matching or deterministically matched or probabilistic matching or probabilistic mean match$ or probabilistic-mean match$ or probabilistically matched or probabilistic method$ or deterministic method$ or probabilistic linkage or probabilistic-linkage or deterministic linkage or deterministic-linkage or probabilistic link or probabilistic-link or deterministic link or deterministic-link or probabilistic linking or probabilistic-linking or deterministic linking or deterministic-linking).mp.
6. (validate or validated or validation).mp.
7. exp "Sensitivity and Specificity"/

**Search Strategy**

1. 1 or 2 or 3 or 4 or 5 (Population)
2. 6 or 7 or 8 or 9 or 10 (Intervention/Exposure)
3. 11 or 12 or 13 or 14 or 15 or 16 or 17 (Outcome)
4. 18 and 19 and 20 (Population+ Intervention/Exposure+ Outcome

# Table 2. Pregnancy Identification Algorithms - Electronic Health Records

| Study, Country, Publication Year | Sample size (Time-Period) | Algorithm Logic | Start date | End date | Gestational age | Conflict/Overlap Handling | Validation Approach | Validation Metrics |
| --- | --- | --- | --- | --- | --- | --- | --- | --- |
| (1), UK, 2004 | 297,082 pregnancies (1991-1999) | Backward-looking logic. | Earliest pregnancy start marker from outcome date in the preceding 280-days. | Outcome anchored. | Not estimated, assumed from start date estimation. | All valid outcomes flagged, invalid start and end date combinations (>280 days) removed. | Clinical inspection of 200 records to check the proportion of unmapped pregnancies and plausibility of pregnancy start and end dates. | No metrics provided. |
| (2), UK, 2010 | 580,356 pregnancies (1987-2006) | Hierarchical. | Earliest marker from outcome date within 280-day window. | Outcome anchored. | Not estimated, assumed from start date estimation. | Hierarchical de-duplication based on timing and prioritisation. | Agreement measured by comparing to maternity dataset | Live births (99%, Cohen’s Kappa=0.99); spontaneous abortions (77%); stillbirths (98%). |
| (3), UK, 2019 | 405,591 pregnancies (2004-2016) | Hierarchical. | Combination of early-marker and back-calculation | Outcome anchored. | Not estimated, assumed from start date estimation. | Not explicitly stated. | 10% chart review; of women aged 29-years, benchmarking against national trends | No metrics provided. |
| (4), UK, 2019 | 5,824,381 pregnancies (1987-2018) | Hierarchical. | Back-calculation | Outcome anchored. | GA code or imputed based on plausible outcome duration if absent. | Overlapping pregnancies and uncertain episodes flagged. | Linked to hospital/national records. | 91% sensitivity, 88% PPV |
| (5), UK, 2023 | 16,833,427 pregnancies (1987-2021) | Hierarchical. | Back-calculation | Outcome anchored. | GA code or imputed based on plausible outcome duration if absent. | Overlapping pregnancies and uncertain episodes flagged | Proportions compared to CPRD GOLD (4) and hospital/national records. | No metrics provided. |
| (6), USA, 2021 | 63,334 distinct deliveries (2010-2017) | Backward-looking logic. | Back-calculation | Outcome anchored | Not estimated, assumed from start date estimation. | Delivery encounters occurring ≥180 days marked a new delivery episode. | Validation against hospital birth log. | 98.6% accuracy, F1-score of 92.1%, PPV of 98.8%, and sensitivity of 89.6%. |
| (7), USA, 2023 | 816,471 pregnancies (2018-2022) | Hierarchical. | Back-calculation | Outcome anchored | GA codes. | Overlaps merged based on gestational plausibility and reclassified or removed if duration misaligns with outcome. | Clinician validation (n=280) | 98.8% agreement for episode identification and >90% concordance for key dates. |
| (8), USA, 2024 | 18,970 pregnancies (2018-2022) | Hierarchical. | Back-calculation | Outcome anchored | GA codes. | Overlaps merged based on gestational plausibility and reclassified or removed if duration misaligns with outcome. | Survey data comparison | Sensitivity of 81.8%, specificity >99%, PPV of 83.1%, and NPV of 98.5%. |
| (9), USA, 2011 | 2,201 pregnancies (1999-2007) | Forward-looking logic. | Earliest marker. | Outcome anchored | Not calculated. | Not explicitly discussed. | Validation of 605 “non-pregnant” cases | High specificity (<0.5% false negatives). |
| (10), USA, 2007 | 24,680 pregnancies (1998-2001) | Hierarchical. | Back-calculation | Outcome anchored | Derived from discharge data or imputed based on plausible outcome duration if missing. | Overlapping and inconsistent pregnancies noted but not discussed how dealt with. | Validation against 511 chart-reviewed cases | 98% outcome date, 99% live births, 91-98% gestational age within 4 weeks. |
| (11), USA, 2011 | 2,173 pregnancies (2009 only) | Event-based | Not explicitly defined. | Outcome anchored | Not estimated. | Not explicitly discussed. | Validation against EHR review. | Sensitivity 94.1-96.0%, PPV between 94.4-98.3%. |

***Abbreviations****: USA: United States of America; UK: United Kingdom; GA: Gestational Age; PPV: Positive Predictive Value; NPV: Negative Predictive Value.*

# Table 3. Pregnancy Identification Algorithms - Claims Data

| Study, Country | Sample size | Algorithm Logic | Start date | End date | Gestational age | Conflict/Overlap Handling | Validation Approach | Validation Metrics |
| --- | --- | --- | --- | --- | --- | --- | --- | --- |
| (12), USA, 2022 | 1,030,874 pregnancies (2015-2020) | Hierarchical. | Back-calculation | Outcome anchored | GA codes or assigned based on plausible outcome duration. | Pregnancies were considered distinct if separated by at least 24 weeks (for live births) or 6 weeks (for losses), and nested or ambiguous episodes were removed | Not conducted. | Not applicable. |
| (13), USA, 2023 | 53,956 pregnancies (2016-2017) | Six algorithms compared. | Back-calculation | Outcome anchored | GA codes or assigned based on plausible outcome duration. | Overlapping pregnancies were excluded. | Chart review of 365 pregnancy outcomes to determine the best performing algorithm for each | Spontaneous abortion (Algorithm 1) PPV: 84.7; Preeclampsia (Algorithm 5) PPV 85.7; Premature delivery (Algorithm 1) PPV 92.3; Low birthweight (Algorithm 1) PPV 96.3. |
| (14), USA, 2021 | 34,204 pregnancies (2016-2018) | Hierarchical. | Back-calculation | Outcome anchored | GA codes, ultrasound timing, or proxy prenatal test | Conflicting outcome codes were excluded. | 284 Physician adjudication | 100% for live births and spontaneous abortions, 71% for stillbirths. GA estimates within ±7 days of the reference in 85.9% of full-term, 81.7% of preterm births, 61.3% of spontaneous abortions and 66.7% of stillbirths. |
| (15), USA, 2019 | 2,978,707 pregnancies (2011-2015) | Hierarchical. | Back-calculation | Outcome anchored | GA codes or assigned based on plausible outcome duration. | Spacing rules to prevent overlap, and pregnancy outcome codes occurring within 30 days grouped. | Compared spontaneous abortion and stillbirth rates to national statistics. | No metrics provided. |
| (16), USA, 2020 | 7,060,675 pregnancies (2005-2018) | Hierarchical | Back-calculation | Outcome anchored | GA codes or assigned based on plausible outcome duration. | Removed outpatient claims for live, mixed, or unclassified delivery ±30 days of an inpatient live birth or delivery claim. | Not conducted. | Not applicable. |
| (17), USA, 2016 | 488,887 pregnancies (2013 only) | Hierarchical | Back-calculation | Outcome anchored | GA codes or assigned based on plausible outcome duration. | Separate pregnancies required a minimum of 2 months between outcome and next estimated LMP to distinguish between episodes. | Compared outcome proportions (e.g., live births, preterm births) to other published datasets for plausibility. | Post-hoc benchmarking found that estimates for antidepressant use and gestational timing were consistent with national statistics. |
| (18), USA, 2023 | 6,520,768 pregnancies (2008-2019) | Hierarchical. | Back-calculation | Outcome anchored | GA codes | Required ≥120 days from a live or stillbirth, and ≥42 days from the end of all other outcomes. | Compared outcome and GA distributions with national patterns. | Not provided. |
| (19), USA, 2023 | 10,162 non-livebirth outcomes (2000-2014) | Event-based. | Back-calculation | Outcome anchored | Not estimated, outcome type inferred likely timing. | Excluded overlapping outcome codes occurring within ±5 days. | Validation against chart-reviewed medical records (n=300; 100 per outcome under primary definitions) | PPV of 94.9% for elective termination, 86.6% for spontaneous abortion, and 80.6% for stillbirth. A composite outcome PPV of 94.4% was observed. |
| (20), USA, 2001 | 3,267 women (1993-1994) | Forward-looking logic | Earliest pregnancy start marker. | Outcome anchored | Not estimated. | Not described. | Chart review of 600 women | 99% agreement among those with markers and outcomes, and 27% for those with markers but no coded outcomes. |
| (21), USA, 2021 | 1,927,071 pregnancies across two databases (2014-2016) | Hierarchical. | Back-calculation | Outcome anchored | GA codes | Hierarchically resolved. | Compared to national benchmarks. | Not provided. |
| (22), USA, 2025 | 4,084,474 deliveries (2006-2021) | Hierarchical. | Back-calculation | Outcome anchored | GA codes, or prenatal screening proxies if missing | A 90-day interval between delivery dates enforced to ensure distinct episodes. | Not conducted. | Not applicable. |
| (23), France, 2018 | 9,647,843 pregnancies (2007-2014) | Backward-looking logic. | Back-calculation | Outcome anchored | GA codes or imputed when missing. | Spacing rules of ≥28 weeks post-live birth and ≥6 weeks post-loss were enforced. | Comparisons with official 2014 birth statistics | Near-complete ascertainment (0.05% difference), and pregnancy start dates aligned within one week of maternity leave-based conception dates in 97.3% of eligible cases. |
| (24), Germany, 2018 | 1,235,261 pregnancies (2006-2014) | Hierarchical. | Back-calculation | Outcome anchored | Not estimated. | Explicitly accounted for date conflicts and implausible sequences, e.g. abortions during pregnancies resulting in live birth, were detected and excluded (n=393). | Expert review of 20 episodes | 100% concordance on outcome classification and 95% on timing. |

***Abbreviations****: USA: United States of America; UK: United Kingdom; GA: Gestational Age; PPV: Positive Predictive Value; NPV: Negative Predictive Value.*

# Table 4. Pregnancy Identification Algorithms - Registry Data and Combination of Health Records

| Study, Country | Sample size | Algorithm Logic | Start date | End date | Gestational age | Conflict/Overlap Handling | Validation Approach | Validation Metrics |
| --- | --- | --- | --- | --- | --- | --- | --- | --- |
| (25), USA, 2013 | 595,929 pregnancies (2002-2006) | Hierarchical. | Back-calculation | Outcome anchored | GA codes or imputed based on plausible outcome duration if missing. | Not explicitly discussed. | Chart review of 420 episodes. | All outcomes 88-100%. Gestational age 36-100% |
| (26), USA, 2021 | 2,485,410 pregnancies (2002-2018) | Hierarchical. | Back-calculation | Outcome anchored | GA codes or imputed based on plausible outcome duration if missing. | Not explicitly discussed. | Validation via chart review (n=375) | 66-100% agreement on outcome type and 73-97% agreement on gestational age |
| (27), U.S. and U.K., 2018 | 5,769,359 pregnancies across four databases in two countries (1987-2013) | Hierarchical. | Back-calculation | Outcome anchored | GA codes, earliest pregnancy marker, or imputed based on plausible outcome. | Episodes were only retained if the start and end date were biologically plausible and outcome specific timings were met. | Validation against 700 patient profiles. | 99-100% accuracy in status and outcome, 95-100% agreement on outcome date, 90-97% agreement on start date. |
| (28), Norway, 2024 | 649,703 pregnancies (2008-2018) | Hierarchical. | Back-calculation | Outcome anchored | Inferred using start marker codes with known GA or imputed based on plausible outcome. | Not stated. | Expert review of 20 early loss cases and registry agreement metrics of miscarriage across primary and secondary care | Primary care - Cohen’s Kappa: 0.93 (0.93-0.93); PPV: 99.2, NPV: 98.4; Agreement: 98.5% (93.8-99.1%)  Secondary care: Cohen’s Kappa - 0.82 (0.82-0.82); PPV: 99.8, NPV: 96.1; Agreement: 96.4% (84.2-98.0%). |
| (29), New Zealand, 2018 | 941,468 pregnancies (2005-2015) | Backward-looking logic. | Back-calculation | Outcome anchored | Estimated based on plausible outcome duration. | Admissions with pregnancy outcome codes occurring within 6 weeks of each other were considered to relate to one pregnancy. | Internal consistency across datasets and national coverage supported the reliability of the cohort. | Not reported. |
| (30), United Kingdom, 2025 | 266,758 women with 279,027 pregnancies (2020-2022) | Hierarchical | Back-calculation | Outcome anchored | GA code or imputed based on plausible outcome duration if absent. | Overlapping pregnancies and uncertain episodes flagged. | Linked to hospital/national records. | 92-98% PPV |
| (31), Korea,  2024 | 3,513 women with 5,800 pregnancy episodes (2005-2018) | Hierarchical | Back-calculation | Outcome anchored | GA code or imputed based on plausible outcome duration if absent. | Not stated. | Not conducted. | Not applicable. |

***Abbreviations****: USA: United States of America; UK: United Kingdom; GA: Gestational Age; PPV: Positive Predictive Value; NPV: Negative Predictive Value*

# References

1. Hardy JR, Holford TR, Hall GC, Bracken MB. Strategies for identifying pregnancies in the automated medial records of the General Practice Research Database. Pharmacoepidemiology and Drug Safety. 2004;13:749-59.

2. Devine S, West S, Andrews E, Tennis P, Hammad TA, Eaton S, et al. The identification of pregnancies within the general practice research database. Pharmacoepidemiology & Drug Safety. 2010;19(1):45-50.

3. Liyanage H, Williams J, Byford R, de Lusignan S. Ontology to identify pregnant women in electronic health records: primary care sentinel network database study. BMJ Health & Care Informatics. 2019;26(1):e100013.

4. Minassian C, Williams R, Meeraus WH, Smeeth L, Campbell OMR, Thomas SL. Methods to generate and validate a Pregnancy Register in the UK Clinical Practice Research Datalink primary care database. Pharmacoepidemiology & Drug Safety. 2019;28(7):923-33.

5. Campbell J, Shepherd H, Welburn S, Barnett R, Oyinlola J, Oues N, Williams R. Methods to refine and extend a Pregnancy Register in the UK Clinical Practice Research Datalink primary care databases. Pharmacoepidemiology & Drug Safety. 2023;32(6):617-24.

6. Canelon SP, Burris HH, Levine LD, Boland MR. Development and evaluation of MADDIE: Method to Acquire Delivery Date Information from Electronic health records. International Journal of Medical Informatics. 2021;145:104339.

7. Jones SE, Bradwell KR, Chan LE, McMurry JA, Olson-Chen C, Tarleton J, et al. Who is pregnant? Defining real-world data-based pregnancy episodes in the National COVID Cohort Collaborative (N3C). JAMIA Open. 2023;6.

8. Smith LH, Wang W, Keefe-Oates B. Pregnancy episodes in All of Us: harnessing multi-source data for pregnancy-related research. Journal of the American Medical Informatics Association. 2024;31(12):2789-99.

9. Strom BL, Schinnar R, Jones J, Bilker WB, Weiner MG, Hennessy S, et al. Detecting pregnancy use of non-hormonal category X medications in electronic medical records. J Am Med Inform Assoc. 2011;18:i81-6.

10. Hornbrook MC, Whitlock EP, Berg CJ, Callaghan WM, Bachman DJ, Gold R, et al. Development of an algorithm to identify pregnancy episodes in an integrated health care delivery system. Health Services Research. 2007;42(2):908-27.

11. Penman-Aguilar A, Tucker MJ, Groom AV, Reilley BA, Klepacki S, Cullen T, et al. Validation of algorithm to identify American Indian/Alaska Native pregnant women at risk from pandemic H1N1 influenza. American Journal of Obstetrics & Gynecology. 2011;204(6):S46-53.

12. Bertoia ML, Phiri K, Clifford CR, Doherty M, Zhou L, Wang LT, et al. Identification of pregnancies and infants within a US commercial healthcare administrative claims database. Pharmacoepidemiology & Drug Safety. 2022;31(8):863-74.

13. Chomistek AK, Phiri K, Doherty MC, Calderbank JF, Chiuve SE, McIlroy BH, et al. Development and Validation of ICD-10-CM-based Algorithms for Date of Last Menstrual Period, Pregnancy Outcomes, and Infant Outcomes. Drug Safety. 2023;46(2):209-22.

14. Moll K, Wong HL, Fingar K, Hobbi S, Sheng M, Burrell TA, et al. Validating Claims-Based Algorithms Determining Pregnancy Outcomes and Gestational Age Using a Linked Claims-Electronic Medical Record Database. Drug Safety. 2021;44(11):1151-64.

15. MacDonald SC, Cohen JM, Panchaud A, McElrath TF, Huybrechts KF, Hernández-Díaz S. Identifying pregnancies in insurance claims data: Methods and application to retinoid teratogenic surveillance. Pharmacoepidemiol Drug Saf. 2019;28(9):1211-21.

16. Sarayani A, Wang X, Thai TN, Albogami Y, Jeon N, Winterstein AG. Impact of the Transition from ICD-9-CM to ICD-10-CM on the Identification of Pregnancy Episodes in US Health Insurance Claims Data. Clin Epidemiol. 2020;12:1129-38.

17. Ailes EC, Simeone RM, Dawson AL, Petersen EE, Gilboa SM. Using insurance claims data to identify and estimate critical periods in pregnancy: An application to antidepressants. Birth Defects Research Part A: Clinical and Molecular Teratology. 2016;106(11):927-34.

18. Ailes EC, Zhu W, Clark EA, Huang YA, Lampe MA, Kourtis AP, et al. Identification of pregnancies and their outcomes in healthcare claims data, 2008-2019: An algorithm. PLoS ONE [Electronic Resource]. 2023;18(4):e0284893.

19. Zhu Y, Bateman BT, Hernandez-Diaz S, Gray KJ, Straub L, Reimers RM, et al. Validation of claims-based algorithms to identify non-live birth outcomes. Pharmacoepidemiology and Drug Safety. 2023;32:468-74.

20. Manson JM, McFarland B, Weiss S. Use of an automated database to evaluate markers for early detection of pregnancy. American Journal of Epidemiology. 2001;154(2):180-7.

21. Sumner KM, Ehlinger A, Georgiou ME, Wurst KE. Development and evaluation of standardized pregnancy identification and trimester distribution algorithms in U.S. IBM MarketScan R Commercial and Medicaid data. Birth Defects Research. 2021;113(19):1357-67.

22. Kahrs JC, Nickel KB, Wood ME, Dublin S, Durkin MJ, Osmundson SS, et al. Development of a Pregnancy Cohort in Commercial Insurance Claims Data: Evaluation of Deliveries Identified From Inpatient Versus Outpatient Claims. Pharmacoepidemiology & Drug Safety. 2025;34(3):e70115.

23. Blotière PO, Weill A, Dalichampt M, Billionnet C, Mezzarobba M, Raguideau F, et al. Development of an algorithm to identify pregnancy episodes and related outcomes in health care claims databases: An application to antiepileptic drug use in 4.9 million pregnant women in France. Pharmacoepidemiol Drug Saf. 2018;27(7):763-70.

24. Wentzell N, Schink T, Haug U, Ulrich S, Niemeyer M, Mikolajczyk R. Optimizing an algorithm for the identification and classification of pregnancy outcomes in German claims data. Pharmacoepidemiology and Drug Safety. 2018;27(9):1005-10.

25. Naleway AL, Gold R, Kurosky S, Riedlinger K, Henninger ML, Nordin JD, et al. Identifying pregnancy episodes, outcomes, and mother-infant pairs in the Vaccine Safety Datalink. Vaccine. 2013;31(27):2898-903.

26. Naleway AL, Crane B, Irving SA, Bachman D, Vesco KK, Daley MF, et al. Vaccine Safety Datalink infrastructure enhancements for evaluating the safety of maternal vaccination. Therapeutic Advances in Drug Safety. 2021;12.

27. Matcho A, Ryan P, Fife D, Gifkins D, Knoll C, Friedman A. Inferring pregnancy episodes and outcomes within a network of observational databases. PLoS ONE [Electronic Resource]. 2018;13(2):e0192033.

28. Nordeng H, Lupattelli A, Engjom H, M.,, van Gelder MHJ. Detecting and Dating Early Non-live Pregnancy Outcomes: Generation of a Novel Pregnancy Algorithm From Norwegian Linked Health Registries. Pharmacoepidemiology and Drug Safety. 2024;33(9):e70002.

29. Donald S, Barson D, Parkin L, Horsburgh S, Sharples K. Generation of a New Zealand pregnancy cohort for medicine utilisation and safety studies. Pharmacoepidemiology and Drug Safety. 2018;27:244-5.

30. Snelling AJHL, Copland E, Mei WX, Mtika WM, Ranger T, Coupland C, et al. Methods to establish a Pregnancy Register in the QResearch Database. Communications Medicine. 2025;5(1):528.

31. Jung YS, Song YJ, Keum J, Lee JW, Jang EJ, Cho SK, et al. Identifying pregnancy episodes and estimating the last menstrual period using an administrative database in Korea: an application to patients with systemic lupus erythematosus. Epidemiol Health. 2024;46:e2024012.
